# Prevalence of hypertension and associated factors in people living with HIV at Senkatana Clinic Maseru

**DOI:** 10.4102/phcfm.v17i1.4813

**Published:** 2025-07-22

**Authors:** Mosa S.M. Selebalo, Benjamin J. Bryden, David M. Thompson, Jill E. Sanders

**Affiliations:** 1 Department of Family Medicine, Family Medicine Specialty Training program, Lesotho Boston Health Alliance, Maseru, Lesotho; 2 General Practice, Ministry of Health, Maseru, Lesotho; 3 Department of Family Medicine, Faculty of Health Sciences, University of Montana, Missoula, United States of America; 4 Department of Family Medicine, Faculty of Health Sciences, University of Washington, Seattle, United States of America; 5 Department of Biostatistics and Epidemiology, Emeritus Faculty, University of Oklahoma Health Sciences, Oklahoma City, United States of America; 6 Department of Family Medicine, Family Medicine Specialty Training Program, Lesotho Boston Health Alliance, Maseru, Lesotho; 7 Department of Pediatrics, Ministry of Health, Maseru, Lesotho

**Keywords:** hypertension, prevalence, antiretroviral therapy, people living with HIV, cardiovascular diseases, healthcare delivery

## Abstract

**Background:**

Cardiovascular diseases (CVDs), including hypertension (HTN), have emerged among people living with HIV (PLHIV) as the most important prevalent contributors of non-AIDS-related mortality. Moreover, HTN itself is a modifiable risk factor for other CVDs. Data are limited regarding the prevalence of HTN and associated factors among PLHIV in Lesotho.

**Aim:**

To determine the prevalence of HTN and associated factors among PLHIV attending Senkatana ART Clinic.

**Setting:**

The study was carried out at Senkatana ART Clinic in urban Maseru, Lesotho.

**Methods:**

A cross-sectional study was conducted from February to July 2022. Data were analysed using SAS statistical software (v9.4) and Microsoft Excel. To determine HTN prevalence, blood pressure (BP) was measured, and a questionnaire was administered to participants who were randomly selected using the lottery method from the clinic’s daily attendance list. Multiple logistic regression was used to assess factors associated with HTN in PLHIV while controlling for potentially confounding factors.

**Results:**

The prevalence of HTN was 57% (exact 95% CI: 51.2%, 62.7%). Of those with HTN, 33.3% were newly diagnosed during this study (exact 95% CI: 26.3%, 40.9%), while 69% of those previously diagnosed with HTN had uncontrolled HTN at enrollment (exact 95% CI: 60.0%, 77.6%). Age older than 50 years (*p* < 0.0001) and a body mass index (BMI) of 25.0 kg/m^2^ or higher (*p* < 0.0002) were independently associated with HTN.

**Conclusion:**

Hypertension was highly prevalent and poorly controlled. Factors associated with HTN in PLHIV were older age and higher BMI. The study’s findings support models of comprehensive healthcare delivery.

## Introduction

There is a growing burden of non-communicable diseases (NCDs) among people living with HIV (PLHIV), particularly in resource-limited health systems of the sub-Saharan Africa, which also bear high burdens of human immunodeficiency virus (HIV) prevalence.^1,2^ Hypertension (HTN) is a major modifiable risk factor for other cardiovascular diseases and has emerged as among the most important and prevalent contributors of non-acquired immunodeficiency syndrome (AIDS)-related mortality.^3,4,5^ Hypertension is responsible for nearly 20% of all global mortality and 9% of global disability-adjusted life years.^6^ Approximately one in four adults in the world have HTN, and Africa is a World Health Organization (WHO) region with the highest prevalence among adults, with some studies estimating 46% of the African population over 25 years of age to be hypertensive and with a steady or increasing overall trend.^7^

Global prevalence of HTN in PLHIV on antiretroviral therapy (ART) was estimated to be 35%.^8^ This was found to be higher in PLHIV over 50 years of age – 42% globally and up to 45.9% in low- and middle-income countries (LMICs).^2,9^ In Western Cape province, South Africa, the crude prevalence of HTN in PLHIV was 41.2% and higher, at 53%, in rural districts of the Eastern Cape.^10,11^ The high prevalence is a serious problem because HTN is an important predictor of long-term survival in PLHIV.^12^ It has been found that PLHIV with HTN at the time of their diagnosis have more than twice the hazard of early mortality compared to those without HTN, which worsens if HTN is undiagnosed and hence untreated.^12^ Furthermore, PLHIV on HAART are at an increased risk (two times higher) of acquiring HTN than HIV-uninfected counterparts and even at higher risk of HTN-related complications. This risk further increases for PLHIV on HAART as opposed to HAART-naive patients. Undiagnosed HTN further increases the risk of HTN-related complications.^3,4,5^

People living with HIV are at higher risk of HTN because of low CD4 count, high viral burden, prolonged exposure to HAART and longer duration of HIV infection. This may be explained by association with HTN-related mechanisms such as renin-angiotensin activation, long-term immune suppression, chronic inflammation and endothelial dysfunction. Some side effects of highly active antiretroviral therapy (HAART), include dyslipidemia, lipodystrophy and nephrotoxicity, which contribute to increase in blood pressure (BP). People living with HIV are also subject to risk factors for HTN, including older age, smoking, high BMI and positive family history of HTN, which are known for people who are not infected with HIV.^4,5,8,13^

Approximately 22.7% of the Lesotho population, a Southern African LMIC, aged 15 years and above live with HIV according to the Lesotho Population-Based HIV Impact Assessment 2020.^14^ The HTN prevalence in the general population in Lesotho was estimated to be 22% in adults.^15^ However, the prevalence of HTN among PLHIV in Lesotho is unknown, as are demographic and clinical characteristics that may be associated with HTN. Therefore, this study aims to determine the prevalence of HTN and associated factors among PLHIV attending Senkatana ART Clinic, Maseru, Lesotho.

## Research methods and design

### Study design

A cross-sectional study was conducted from February to July 2022 at Senkatana ART Clinic, Maseru, Lesotho. Analytical cross-sectional design is used, as it can determine the prevalence of a condition and association with several factors, at a given point in time, without establishing risks, causality or a cause-effect between exposure and outcome.^16,17^ Therefore, it aligns well with the study aim.

### Study setting

Senkatana ART Clinic is found in Maseru, the capital district of Lesotho. Lesotho is a landlocked country surrounded by the Republic of South Africa. It has a total population of 2 135 000, with approximately 25% residing in the Maseru district. Senkatana ART Clinic primarily serves a total population of 23 120 from nine villages in Maseru. All are HIV-positive adults receiving HAART from this centre. It also serves as a national referral centre for special HIV and TB cases. It provides HIV services comprising of prevention, counselling, diagnosis, treatment and monitoring. It follows the test and treat criteria regardless of CD4 cell count.

### Study population, sample and sampling strategy

Participants randomly selected from among PLHIV who attended the clinic were aged 18 years and above, were on HAART for at least 12 months and provided informed consent. Excluded from the study were pregnant patients because of the complexity of hypertensive disease in pregnancy and all those unwilling to provide consent or severely ill. SAS PROC POWER version 9.4 (SAS Institute Inc.) was used to determine that a sample size of 300, drawn from a total clinic population of 23 120, would ensure, with a probability of at least 0.8, that the 95% confidence interval for the study estimates of the prevalence of HTN would have a half-width no larger than 0.05.^18^ The calculation assumed that the true prevalence of HTN in the clinic population was in accordance with the WHO’s most recent estimate for Lesotho, published in 2018, of 0.22.^15^

### Data collection tool and procedure

During patients’ follow-up visits to the clinic, study participants were randomly selected using the lottery method from the attendance list. They were then invited to participate in the study and provide informed consent. Structured questionnaires were used to collect data on socio-demographic, behavioural, medical and family history. Family history of HTN was considered positive for individuals with first-degree relatives known to have HTN. Participants were categorised as smokers if they smoked at least one cigarette per week in the last 12 months, and as alcohol consumers if they consumed any alcoholic drink at least seven times per week during the last 12 months. Clinical data (including duration of HIV Infection and HAART regimen) were collected from both the patients and their clinical records, including WHO clinical staging I, II, III and IV.

Anthropometric data (weight in kg and height in meters) were measured during the interview, and BMI was calculated (kg/m^2^). Body mass index was classified as underweight (BMI < 18.5 kg/m^2^), normal (BMI 18.5 kg/m^2^ – 24.9 kg/m^2^), overweight (25 kg/m^2^ – 29.9 kg/m^2^) and obese (BMI ≥ 30 kg/m^2^). Systolic and diastolic BP were measured on the left arm twice at intervals of at least 5 min, with the patient remaining seated for at least 15 min before the first measurement. The average of the two measurements was recorded in mmHg. Hypertension was defined as either self-reported use of antihypertensive medication or elevated systolic BP of ≥ 140 mmHg or diastolic BP ≥ 90 mmHg. Uncontrolled HTN was defined as either systolic BP of > 140 mmHg or diastolic BP of > 90 mmHg in participants already on anti-HTN medication.

### Data analysis

Data were analysed using SAS statistical software (v9.4) and Microsoft Excel. Hypertension prevalence was calculated as the number of participants qualifying as having HTN divided by the total number sampled. The percentage of undiagnosed HTN was calculated as the number of participants newly diagnosed during the study divided by the total number qualifying as having HTN. Categorical variables (sex, family history of HTN, duration on HAART, age, duration of HIV infection, HAART regimen, WHO clinical stage, smoking status, BMI, alcohol use, educational status, marital status) were summarised using counts (frequencies and percentages/proportions). Continuous variables (age, BMI) were summarised as means and standard deviations. To identify potential factors associated with HTN, participants with and without HTN were compared with respect to each factor in separate bivariable analyses, using exact Pearson Chi-square tests.

To address potential confounding in the identification of associated factors, the study excluded pregnant women and people who were severely ill. Otherwise, the study included anyone living with HIV who was 18 years or older and had been on HAART for at least 12 months. This relatively broad inclusion criteria did not restrict participation on the basis of several known risk factors. Accordingly, multivariable logistic regression models were used to assess potential confounding in the bivariable comparisons.

Hypertension status was the dependent variable in multivariable models constructed through an investigator-mediated forward selection process that regarded a *p*-value of < 0.05 as evidence of statistical significance. The initial multivariable model included, as independent variables, three known risk factors for HTN: age, BMI status and sex. This model found that older age (*p* < 0.0001) and BMI of 25.0 kg/m^2^ or higher (*p* = 0.0002) were independently associated with HTN, but sex, after controlling for age and BMI status, was not (*p* = 0.3148). Based on this finding, each factor’s association with the dependent variable, HTN status, was then assessed in separate multivariable logistic regression models that controlled only for age (in years) and BMI status.

To determine whether a given factor modified the association between HTN and either age or BMI status, that factor’s regression model included two-factor and three-factor interaction terms. For factors for which no statistical interactions were detected, the interaction terms were then removed, and results from a multivariable model that controlled only for age (in years) and BMI status were reported (Table 3). Results for the single factor for which the presence of a statistical interaction indicated that the factor modified the association between HTN and age are reported. For all analyses, a *p*-value of < 0.05 was considered evidence of statistical significance. Analyses were performed using SAS statistical software (v9.4).

### Ethical considerations

Ethical clearance to conduct this study was obtained from the Ministry of Health Lesotho, National Health Research Ethics Committee (NREC) (No. ID 171-2021), as well as from the clinic management. Before participation, the purpose of the study, NREC safeguards and written informed consent were explained to each study participant. The information obtained from participants was the minimum necessity to conduct the study, and interactions occurred in private settings. The confidentiality of the data was protected by assigning a unique study number to each participant. Data collection questionnaires were translated into Sesotho for participants who did not understand English.

## Results

### Demographic characteristics of the study participants

Demographic characteristics are presented in Table 1. A total of 300 participants were included in the study analysis, of whom 63.7% were female. The mean (SD) age was 51.6 years (10.8 years) and 55.7% of the participants were aged 50 years and above. Eighty-seven (29.0%) participants had a positive family history of HTN (first-degree relative), 162 (54%) were married and 161 (53.7%) had not attended school beyond grade 8. Only 48 (16.0%) of the participants were smokers and 62 (20.7%) consumed alcohol.

**TABLE 1:** Demographic characteristics of study participants.

Variables	Category	Frequency	%
Sex	Female	191	63.7
	Male	109	36.3
Age (years)	20–49	133	44.3
	≥ 50	167	55.7
Smoking status	Yes	48	16.0
	No	252	84.0
Alcohol use†	Yes	62	21.4
	No	228	78.6
Family history of HTN	Yes	87	29.0
	No	213	71.0
Marital status	Single	27	9.0
	Married	162	54.0
	Widowed	88	29.3
	Divorced	23	7.7
Educational status	Grade 1–8	161	53.7
	Grade 9–12	121	40.3
	College and above	18	6.0

HTN, hypertension.

† , In the section of alcohol users, all the first 10 responses received were NO, because of misunderstanding of the questionnaire and therefore were discarded and the total participants for this section reduced to 290.

### Clinical characteristics

The mean (SD) BMI was 28.1 kg/m^2^ (6.5 kg/m^2^), and the majority (64.6%) were overweight or obese. A total of 101 (34%) were classified as WHO clinical stage 1. All participants were on HAART, and the most frequently used regimen (76%) was TDF/3TC/DTG. A total of 57.3% had HIV infection duration of more than 10 years, and 57% had received HAART for a similar duration (Table 2).

**TABLE 2:** Clinical characteristics of study participants.

Variables	Categories	Frequency	% R1.10
BMI (kg/m^2^)	< 25.0	106	35.3
	≥ 25.0	194	64.7
Duration of HIV infection (years)	< 5	19	6.3
	5–10	109	36.3
	> 10	172	57.3
Duration on HAART (years)	< 5	20	6.7
	5–10	109	36.3
	> 10	171	57.0
HAART regimen	TDF/3TC/DTG	228	76.0
	ABC/3TC/DTG	37	12.3
	AZT/3TC/DTG	14	4.7
	AZT/3TC/LPV/r	7	2.3
	ABC/3TC/LPV/r	8	2.7
	TDF/3TC/LPV/r	5	1.7
	TDF/3TC/ATV/r	1	0.3
WHO clinical stage	1	101	34.0
	2	55	18.0
	3	133	44.0
	4	11	4.0

WHO, World Health Organization; BMI, body mass index; HIV, human immunodeficiency virus; HAART, highly active antiretroviral therapy; TDF, tenofovir; TC, lamivudine; DTG, dolutegravir; ABC, abacavir; AZT, zidovudine; LPV/r, lopinavir/ritonavir.

### Prevalence of hypertension

The prevalence of HTN was found to be 57% (171/300) (95% CI: 51.2%, 62.7%). Of the 171 participants with HTN, 57 (33.3%) were newly diagnosed during this study (exact 95% CI: 26.3%, 40.9%). Of the 114 participants who were already on HTN treatment, 79 (69.3%) had uncontrolled HTN (exact 95% CI: 60.0%, 77.6%).

### Demographic and clinical factors associated with hypertension in people living with HIV

Hypertension prevalence among people older than 50 years (73.1%) was higher than among younger participants (*p* from exact Chi-square test <0.0001). The overweight and obese group, BMI ≥ 25.0 kg/m^2^, was observed to have a higher prevalence of HTN at 63.9% (*p* from exact Chi-square test = 0.0015) as compared to the normal and underweight group with a HTN prevalence of 44.3%.

**FIGURE 1:**
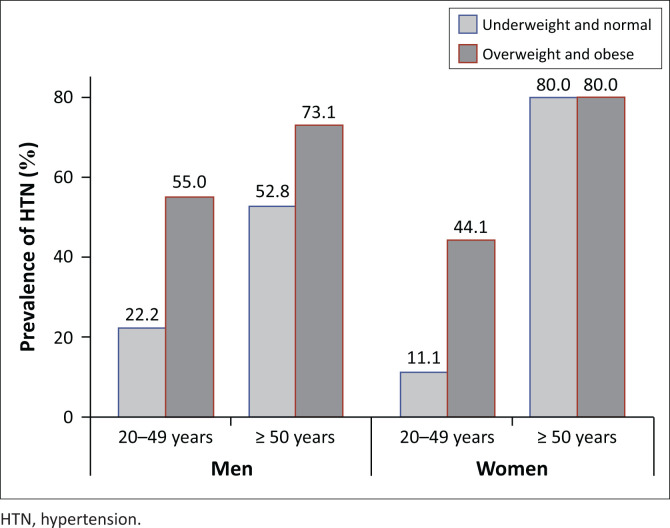
The association between age group and HTN differed between men and women.

Sex was not independently associated with HTN after controlling for age and BMI status. The association between age group and HTN differed between men and women (*p* for sex*age interaction = 0.0237). For men with a given BMI status, the odds of HTN were 3.05 times higher (95% CI: 1.34, 6.94; *p* = 0.0079) among those who are fifty years of age or older, than among those who are younger than 50. For women in a given BMI status, the odds of HTN are 7.19 times higher (95% CI: 3.70,13.97; *p* < 0.001) among those who are fifty years of age or older, than among those who are younger than 50 (Figure 1). In a given age category, the odds of HTN were 2.80 times higher (95% CI: 1.63, 4.82) among those whose BMI of 25.0 kg/m^2^ or higher than among those whose BMI is less than 25.0 kg/m^2^.

The significant bivariable association between HTN and marital status (*p* from exact Chi-square test 0.0007) reflects a higher prevalence of HTN among the widowed (73.9%) and divorced (60.9%) participants, who are older on average than the other groups. In the multivariable analyses, married participants were, on average, older than those who were single, and those who were divorced were not older than those who were married. Widowed participants were older than those in other marital groups. At a given age (in years), HTN was not associated with marital status (*p* = 0.3550). The significant bivariable association between HTN and marital status was confounded by between-group differences in mean age (Table 3).

**TABLE 3:** Bivariable and multivariable analyses of factors associated with hypertension.

Variables	Hypertension				Bivariable analysis (exact Chi-square tests)	Multivariable analysis (logistic regression models that control for age [in years] and BMI status)		
	Yes (*N* = 171)		No (*N* = 129)		*p*-value	*p*-value	Adjusted odds ratio	95% CI
	*n*	%	*n*	%				
**Age (years)**								
20–49	49	36.8	84	63.2	< 0.0001	<0.0001		-
≥ 50	122	73.1	45	26.9	-	-	**	-
**Smoking status**								
Yes	23	47.9	25	52.1	0.2032	0.5644	0.81	0.40, 1.65
No	148	58.7	104	41.3	-	-	Reference	-
**Alcohol use**								
Yes	31	50.0	31	50.0	0.2495	0.9923	1.00	0.53, 1.87
No	140	58.8	98	41.2			Reference	-
**Family history of HTN**								
Yes	51	58.6	36	41.4	0.7974	0.6393	1.15	0.65, 2.03
No	120	56.3	93	43.7	-	-	Reference	-
**Marital status**								
Single	11	40.7	16	59.3	0.0007	0.3550	Reference	-
Married	81	50.0	81	50.0	-	-	0.84	0.33, 2.10
Widowed	65	73.9	23	26.1	-	-	1.26	0.45, 3.58
Divorced	14	60.9	9	39.1	-	-	1.75	0.51, 6.06
**Educational status**								
Grade 1–8	93	60.3	64	39.8	0.1068	0.7294	Reference	-
Grade 9–12	61	50.4	60	49.6	-	-	0.87	0.50, 1.51
College and above	13	72.2	5	27.8	-	-	1.34	0.42, 4.30
**BMI status**								
< 25.0 kg/m^2^	47	44.3	59	55.7	0.0015	0.0002	Reference	-
≥ 25.0 kg/m^2^	124	63.9	70	36.1	-	-	2.80	1.63, 4.82
**Duration of HIV infection (years)**								
< 5	9	47.4	10	52.6	0.2409	0.8334	Reference	-
5–10	57	52.3	52	47.7	-	-	0.71	0.23, 2.22
> 10	105	61.0	67	39.0	-	-	0.72	0.23, 2.21
**Duration on HAART (years)**								
< 5	10	50.0	10	50.0	0.3162	0.7966	Reference	-
5–10	57	52.3	52	47.7	-	-	0.69	0.22, 2.12
> 10	104	60.8	67	39.2	-	-	0.69	0.23, 2.09
**HAART regimen**								
TDF/3TC/DTG	125	54.8	103	45.2	0.0588	0.3411	Reference	
ABC/3TC/DTG	29	78.4	8	21.6	-	-	2.27	0.91, 5.65
AZT/3TC/DTG	7	50.0	7	50.0	-	-	0.49	0.15, 1.55
AZT/3TC/LPV/r	2	28.6	5	71.4	-	-	0.33	0.05, 2.05
ABC/3TC/LPV/r	5	62.5	3	37.5	-	-	1.43	0.29, 7.18
TDF/3TC/LPV/r	3	60.0	2	40.0	-	-	1.38	0.16, 11.87
TDF/3TC/ATV/r	0	0.0	1	100.0	-	-	Not estimable	-
**WHO clinical stage**								
1	60	59.4	41	40.6	0.2153	0.3365	Reference	-
2	37	67.3	18	32.7	-	-	1.00	0.46, 2.14
3	68	51.1	65	48.9	-	-	0.61	0.33, 1.11
4	6	54.5	5	45.5	-	-	0.78	0.19, 3.15

HTN, hypertension; WHO, World Health Organization; HIV, human immunodeficiency virus; highly active antiretroviral therapy; TDF, tenofovir; TC, lamivudine; DTG, dolutegravir; ABC, abacavir; AZT, zidovudine; LPV/r, lopinavir/ritonavir.

** , Separate odds ratios by age category for men and women are reported earlier.

The bivariable association between HTN and HAART regiment approached significance (*p* = 0.0588). The prevalence of HTN was found highest in the subgroup receiving ABC/3TC/DTG. However, in multiple logistic regression, the bivariable association between HTN and HAART regimen was found to be confounded and explained by differences in age among the groups undergoing different regimens (Table 3).

## Discussion

The aim of this study was to determine the prevalence of HTN and associated factors among PLHIV attending Senkatana ART Clinic, Maseru, Lesotho. The prevalence of HTN was found to be high (57%), with a third of the participants newly diagnosed during the study. This prevalence is comparable to the 53% reported in rural districts of the Eastern Cape, South Africa.^11^ It is also similar to the prevalence reported for other LMICs (45.9%) and in South Africa (50.1%) among adults over 50 years of age on HAART,^9,19^ but higher than the global estimate of 35% HTN prevalence among PLHIV on HAART.^8^ It is also higher than that reported for the general population of Lesotho (22%).^15^ It is not very much higher than 41.2% reported in Western Cape, South Africa.^10^

The mean age of participants in this study (51.6 years) is higher than mean age of 38.4 years in a similar study conducted in the Western Cape. This population’s relatively high prevalence of HTN could be attributed to demographic and clinical characteristics of the study’s sample, including a higher BMI status (63.9%) and older age. It could also be because of differing sampling processes, as in the Western Cape, participants were selected from 62 different health facilities, had a lower mean age (38.4 years) and lower proportion of overweight (43.9%) participants.^10,20^

Among participants previously on antihypertensive medication, more than half had uncontrolled HTN. This rate was higher than the 25.9% reported in the Western Cape, South Africa, but comparable to the 58.2% reported in a prospective cohort from a public ART clinic in South Africa.^10,20^ About one-third of the participants were newly diagnosed during the study, which is a relatively lower proportion than reported in Northeast Ethiopia (74.4 %) but comparable to the 50% reported for the general population.^6,8^ This may point towards missed opportunities to adequately diagnose and manage HTN in patients who are regularly in contact with the healthcare system. Matters of non-adherence to anti-HTN medication because of pill burden or other reasons, ineffective choice of medications or vertical health programmes can affect control rate. There is need to offer integrated, comprehensive and person-centred healthcare services to PLHIV to ensure early diagnosis, adequate control of HTN and prevention of related complications.^4,13,20^

Evidence of statistical interaction was detected in the multivariable logistic regression model that explored associations between HTN and sex. The interaction between sex and age (*p* = 0.0237) signifies that differences in the prevalence of HTN between men and women differ for older and younger participants. Prevalence is higher among older participants, regardless of sex, but that difference in prevalence is larger for women than for men. This finding is supported by other studies that show that at younger age, males are at higher risk for HTN, but it changes in older age where women are more at risk.^4,8,19,20^ This points towards the need to enhance vigilance for HTN screening and diagnosis as well as care and treatment among women as they age.

This study’s finding that older age and higher BMI were independently associated with HTN is in agreement with other African studies.^8,10,20,21^ This suggests that the same factors affecting the general population also affect PLHIV and highlights the need to offer a more comprehensive approach to healthcare in this population by addressing known modifiable risk factors for HTN, such as maintaining a normal BMI.^3,13^

This study found no association between the prevalence of HTN- and HIV-related factors, such as the duration of infection, the duration of HAART use, HAART regimen and HIV WHO clinical stage. This is contrary to findings in other studies that suggest a significant association between HTN prevalence and HAART regimen, duration of HAART use and of HIV infection, and WHO clinical staging. These studies state that HIV infection leads to endothelial dysfunction because of chronic inflammation, platelet activation and hypercoagulability. Moreover, they state that side effects of HAART put PLHIV at risk for HTN, such as nephrotoxicity and changes in body constitution.^10,11,20,21^

In bivariable analysis, there was a significant difference in prevalence of HTN among marital status groups (*p* value 0.0009). Nonetheless, multivariable analysis suggests that this association is confounded by differences in the groups’ mean ages. However, a study conducted in Benin reported that having a partner reduced the risk of HTN, which could be explained by calming effects of a stable support structure, thereby limiting the effects of stress.^5^

These findings suggest that comorbidities such as HTN are probably contributing to non-AIDS mortalities in this population, as HTN is a well-known risk factor for other NCDs.^3,4^ Early detection and effective management are critical to preventing associated complications. This study shows not only the need for early diagnosis of missed cases but also the importance of addressing poor BP control among those with a known diagnosis.

Recommendation is made to conduct further studies, with different designs including participants from various health facilities to determine the contribution of HIV-related factors to the prevalence of HTN and other NCDs in PLHIV in Lesotho. Therefore, this study is a model for future investigations of their prevalence and association to HIV. These investigations can contribute to the development of optimal clinical practice guidelines for Lesotho.

This study demonstrates an intersection between HIV and HTN. It can be expected that this applies to other NCDs. It is therefore additionally recommended that the health system integrates services for PLHIV to optimise care and make efficient use of resources allocation.

### Strengths and limitations

Generalisability to the greater population of PLHIV may be limited because Senkatana ART is a referral centre for other ART clinics. However, such complicated referred cases are a minority of the general clinic attendees. The study being carrie out in only one ART clinic may not be very representative of the Lesotho general HIV context. The study design offers limitation with regard to causality and risks, as this could not be established; other study designs would need to be implemented towards this goal. Regardless of the questionnaire being in the two local languages, participants still found it difficult to comprehend some questions; hence, mostly the tool was not self-administered, leaving room for information bias.

## Conclusion

People living with HIV are not exempted from NCDs and their complications. Hypertension is highly prevalent among PLHIV, and in a significant percentage of such patients, HTN is either undiagnosed or uncontrolled. Older age and high BMI were found to be independently associated with HTN, and the introduction of routine HTN prevention, diagnostic and treatment measures has the potential to reduce morbidity and mortality within this population and improve the quality of life of patients. There is a need for patient-oriented approach with holistic, comprehensive and integrated medical care rather than siloed, disease-oriented care to address the complexities of PLHIV.
